# Association of COVID-19 Vaccination With Risk for Incident Diabetes After COVID-19 Infection

**DOI:** 10.1001/jamanetworkopen.2022.55965

**Published:** 2023-02-14

**Authors:** Alan C. Kwan, Joseph E. Ebinger, Patrick Botting, Jesse Navarrette, Brian Claggett, Susan Cheng

**Affiliations:** 1 Smidt Heart Institute, Department of Cardiology, Cedars Sinai Medical Center Los Angeles, California; 2 Cardiovascular Division, Brigham and Women’s Hospital, Boston, Massachusetts

## Abstract

This cohort study compares the risk of new-onset hypertension, hyperlipidemia, and diabetes before and after COVID-19 infection among patients who were vaccinated vs unvaccinated before infection.

## Introduction

In early phases of the COVID-19 pandemic, persons who recovered from infection had increased risks for new-onset cardiometabolic diseases, including diabetes, hypertension, and hyperlipidemia.^1,2,3^ In the current pandemic phase, which is dominated by less virulent Omicron variants,^4^ it remains unclear whether risks of cardiometabolic disease after COVID-19 infection persist or have become attenuated and whether vaccination status is associated with these risks.

## Methods

This large cohort study of adult patients with 1 or more COVID-19 infections treated within the Cedars-Sinai Health System in Los Angeles, California from March 2020 to June 2022 used *International Classification of Diseases, Ninth Revision* and *International Statistical Classification of Diseases and Related Health Problems, Tenth Revision* codes (eTable in Supplement 1) to identify cardiometabolic diagnoses (hypertension, hyperlipidemia, and diabetes) newly reported before or after a patient’s first COVID-19 infection.^5^ A self-controlled exposure-crossover design^6^ was used to estimate odds of a new cardiometabolic diagnosis occurring 90 days after vs 90 days before COVID-19 infection. To account for temporal confounders arising from disruptions in health care use during the pandemic, we compared the odds of a new cardiometabolic diagnosis with those of a new benchmark diagnosis (ie, urinary tract infection and gastroesophageal reflux), representing a marker of health care engagement unrelated to COVID-19. In multivariable logistic regression models, we estimated the odds ratio (OR) for a new cardiometabolic vs new benchmark diagnosis occurring 90 days after vs before infection while adjusting for age, sex, timing of infection (before vs after emergence of Omicron variant), and COVID-19 vaccination status (eMethods in Supplement 1). The study was approved by the Cedars Sinai Medical Center Institutional Review Board, which waived the informed consent requirement given the study’s retrospective nature. We followed the STROBE reporting guideline.

Data were analyzed using R, version 4.2.1 (R Foundation for Statistical Computing). The threshold for statistical significance was a 2-tailed *P* <.05.

## Results

The cohort of 23 709 patients (mean [SD] age, 47.4 [19.3] years) included 12 706 females (54%) and 10 981 males (46%) (22 patients of unknown sex) with 1 or more COVID infection. Rates of new-onset diabetes, hypertension, hyperlipidemia, and benchmark diagnoses occurring in the 90 days after COVID-19 infection were higher than those before infection (Figure). The highest odds postinfection were for diabetes (2.35; 95% CI, 1.94-2.89; *P* < .001), followed by hypertension (1.54; 95% CI, 1.35-1.76; *P* < .001), benchmark diagnoses (1.42; 95% CI, 1.25-1.61; *P* < .001), and hyperlipidemia (1.22; 95% CI, 1.03-1.47; *P* = .03). In adjusted multivariable models, risk of new-onset diabetes (vs benchmark) diagnosis occurring after vs before COVID-19 infection was significantly elevated (OR, 1.58; 95% CI, 1.24-2.02; *P* < .001); however, risks of hypertension and hyperlipidemia vs benchmark diagnoses were not (Table). Although the diabetes risk after infection was higher among unvaccinated (OR, 1.78; 95% CI, 1.35-2.37; *P* < .001) than vaccinated (OR, 1.07; 95% CI, 0.64-1.77; *P* = .80) patients, the interaction term between vaccination status and diabetes diagnosis was not statistically significant (OR, 0.59; 95% CI, 0.34-1.06; *P* = .08). There was no evidence of interaction by age, sex, or preexisting cardiovascular risk factors, including hypertension or hyperlipidemia. Age, sex, and timing of index infection regarding the Omicron variant were not associated with an increased risk of a new cardiometabolic diagnosis before or after COVID-19 infection in any model (Table).

**Figure.**
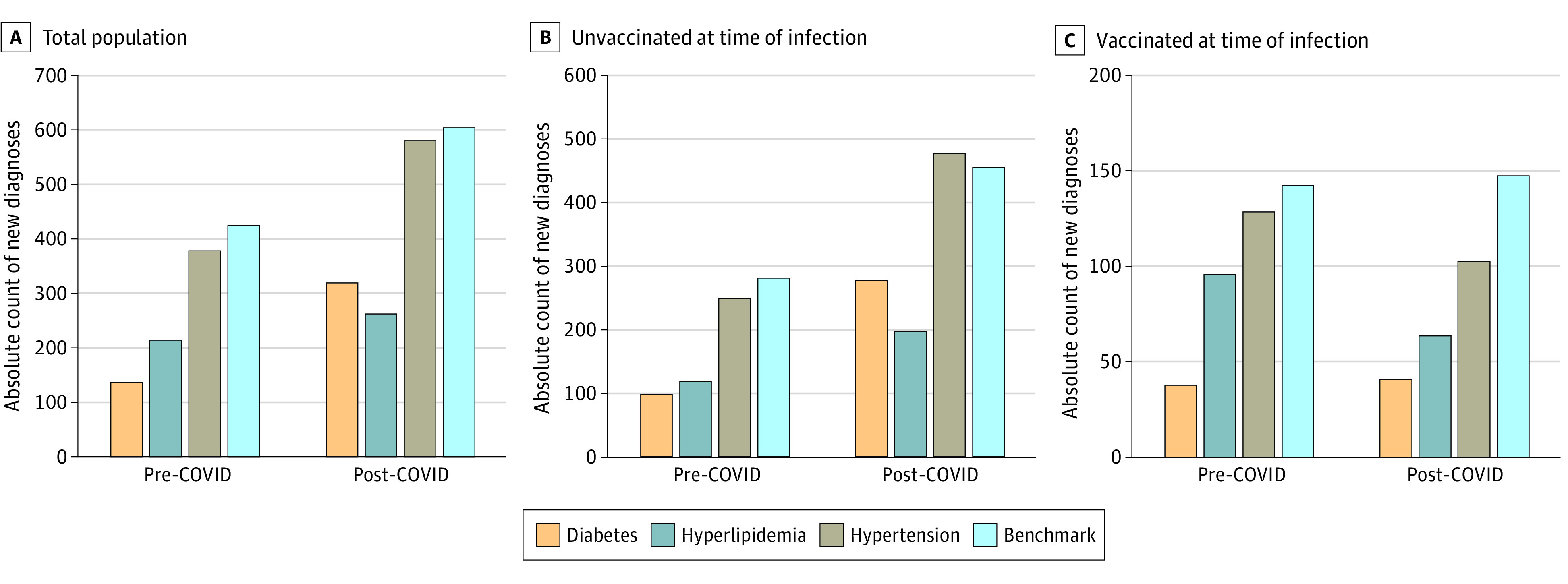
New Diagnoses Before and After COVID-19 Infection The frequency of new diagnoses (diabetes, hyperlipidemia, hypertension, and benchmark conditions) occurring 90 days before and after COVID-19 infection is displayed as color bars in the overall cohort of 23 709 patients (A), in the subset of 14 856 patients who were unvaccinated prior to infection (B), and in the subset of 8853 patients who were vaccinated prior to infection (C).

**Table.** Multivariable-Adjusted Risk for New Cardiometabolic Diagnosis After COVID-19 Infection^a^

Model covariates	New diagnosis post-COVID–19 infection vs pre-COVID–19 infection					
	Diabetes^b^		Hypertension^b^		Hyperlipidemia^b^	
	OR (95% CI)	*P* value	OR (95% CI)	*P* value	OR (95% CI)	*P* value
Age, y	1.00 (0.99-1.01)	.91	1.00 (0.99-1.01)	.77	1.00 (0.99-1.01)	.56
Male sex	0.91 (0.73-1.13)	.39	0.94 (0.78-1.14)	.52	0.83 (0.67-1.03)	.09
Timing of index infection (after vs before emergence of Omicron variant)	0.85 (0.64-1.12)	.24	0.96 (0.76-1.23)	.76	0.99 (0.75-1.30)	.93
Vaccinated vs unvaccinated status before infection	0.63 (0.47-0.85)	.002	0.54 (0.42-0.69)	<.001	0.55 (0.41-0.73)	<.001
New diagnosis of cardiometabolic vs benchmark condition^b^	1.58 (1.24-2.02)	<.001	1.06 (0.88-1.28)	.52	0.91 (0.73-1.15)	.43

Abbreviation: OR, odds ratio.

^a^ Model output is shown for estimating odds ratios for developing new post-COVID diagnosis of a cardiometabolic condition vs benchmark diagnosis adjusting for age, sex, timing of index infection, and pre-infection vaccination status.

^b^ The outcome of each model is defined as the occurrence of a new diagnosis 90 days after vs before COVID-19 infection, with the primary factor being a diagnosis of diabetes, hypertension, or hyperlipidemia vs new diagnosis of a benchmark condition (eg, urinary tract infection or gastroesophageal reflux disease).

## Discussion

In this cohort study, COVID-19 infection was associated with increased risk of diabetes, consistent findings of a meta-analysis.^1^ Our results suggest that this risk persisted as the Omicron variant became predominant, and the association remained even after accounting for temporal confounders. Diabetes risk after COVID-19 infection was higher in unvaccinated than vaccinated patients, suggesting a benefit of vaccination. Mechanisms contributing to postinfection diabetes risk remain unclear, although persistent inflammation contributing to insulin resistance is a proposed pathway. Study limitations include reliance on diagnostic coding, unaccounted confounders (infection severity indices), and insufficient sample size and statistical power for testing multiple interactions. Additional studies are needed to understand cardiometabolic sequelae of COVID-19 and whether COVID-19 vaccination attenuates risk of cardiometabolic disease.

## Notes

Supplement 1.
eMethods.
**eTable.** Outpatient Diagnostic Codes Used for Analyses
eReferences

Supplement 2.
Data Sharing Statement

## References

[1] Zhang T, Mei Q, Zhang Z, . Risk for newly diagnosed diabetes after COVID-19: a systematic review and meta-analysis. BMC Med. 2022;20(1):444. doi:10.1186/s12916-022-02656-y 36380329 PMC9666960

[2] Xie Y, Xu E, Bowe B, Al-Aly Z. Long-term cardiovascular outcomes of COVID-19. Nat Med. 2022;28(3):583-590. doi:10.1038/s41591-022-01689-3 35132265 PMC8938267

[3] Al-Aly Z, Xie Y, Bowe B. High-dimensional characterization of post-acute sequelae of COVID-19. Nature. 2021;594(7862):259-264. doi:10.1038/s41586-021-03553-9 33887749

[4] Karim SSA, Karim QA. Omicron SARS-CoV-2 variant: a new chapter in the COVID-19 pandemic. Lancet. 2021;398(10317):2126-2128. doi:10.1016/S0140-6736(21)02758-6 34871545 PMC8640673

[5] Wei W-Q, Bastarache LA, Carroll RJ, . Evaluating phecodes, clinical classification software, and ICD-9-CM codes for phenome-wide association studies in the electronic health record. PLoS One. 2017;12(7):e0175508. doi:10.1371/journal.pone.0175508 28686612 PMC5501393

[6] Redelmeier DA. The exposure-crossover design is a new method for studying sustained changes in recurrent events. J Clin Epidemiol. 2013;66(9):955-963. doi:10.1016/j.jclinepi.2013.05.003 23850556

