# Supplementary material for: Association of COVID-19 Vaccination With Risk for Incident Diabetes After COVID-19 Infection
Source: JAMA Netw Open. 2023 Feb 14;6(2):e2255965. doi: 10.1001/jamanetworkopen.2022.55965 (PMC9929690; doi:10.1001/jamanetworkopen.2022.55965)
Supplement: Supplement 1. — eMethods. eTable. Outpatient Diagnostic Codes Used for Analyses eReferences [file jamanetwopen-e2255965-s001.pdf]

Supplemental Online Content

Kwan AC, Ebinger JE, Botting P, Navarrette J, Claggett B, Cheng S. Association of COVID-19 vaccination with risk for incident diabetes after COVID-19 infection. *JAMA Netw Open*. 2023;6(2):e2255965. doi:10.1001/jamanetworkopen.2022.55965

eMethods.

eTable. Outpatient Diagnostic Codes Used for Analyses

eReferences

This supplemental material has been provided by the authors to give readers additional information about their work.

© 2023 Kwan AC et al. *JAMA Network Open.*

eMethods

Study Sampling

Of all patients who are cared for as outpatients by the Cedars-Sinai Health System in Los Angeles, we identified adults age ≥18 years who were treated for ≥1 COVID infection within our health system during years 2020 to 2022. The COVID infection event and date were confirmed based on documented presence of a positive diagnostic (e.g. PCR or antigen test) and clinician diagnosis. For any patient who had more than 1 documented infection, we considered first infection as the index infection. We defined any SARS-CoV-2 infection occurring prior to December 15, 2021 as being likely due to a pre-Omicron variant, and any SARS-CoV-2 infection occurring from this date forward was being likely due to an Omicron variant based on surveillance data continually analyzed from water waste and locally collected samples in our region.[1] After using this approach to categorizing the timing of index infections as occurring during the pre-Omicron or Omicron era, we next identified prior infected individuals who attended outpatient visits within the 90 days before and within the 90 days after their index infection. This combined 180-day time window was considered the study period within which we sought to identify new-onset diagnoses of diabetes, hyperlipidemia, hypertension, or 'benchmark' conditions (including urinary tract infection or gastroesophageal reflux disease). Thus, we identified pre-existing diagnoses of these conditions and excluded from the current analysis any individuals who had all 5 of these diagnoses documented >90 days prior to the index COVID infection. As a result, we identified for the current analysis a total study cohort of N=23,709 patients that included 1,919 (8%) with pre-existing diabetes, 4,810 (20%) with pre-existing hypertension, 3,810 (16%) with pre-existing hyperlipidemia, and 4,802 (20%) with pre-existing benchmark diagnoses; in turn, the remaining number of patients at risk for acquiring new diagnoses during the study period included 21,709 for diabetes, 18,899 for hypertension, 19,899 for hyperlipidemia, and 18,907 for benchmark diagnoses. All pre-existing and new-onset diagnoses were determined using

© 2023 Kwan AC et al. *JAMA Network Open.*

outpatient-associated ICD codes (**eTable**).[2] This study follows STROBE guidelines for observational studies.

Study Design

To investigate whether new diagnoses of cardiometabolic conditions (i.e. diabetes, hyperlipidemia, or hypertension) may have occurred more frequently after a COVID infection as opposed to before a COVID infection, we used an exposure-crossover study design that considers study participants as their own control.[3] The exposure-crossover design is intended to control for multiple potential sources of confounding in the setting of an exposure that confers a measurable effect within a relatively short period of time (e.g. an intervention). This study design involves self-matching and then comparing events within a baseline interval prior to the exposure, an induction interval immediately ahead of the exposure, and a subsequent interval after the exposure.[3] In situations where the exposure is not a planned intervention but rather an acute event such as an infection,[4-6] the induction interval is not applicable. Accordingly, for the current study aiming to identify new diagnoses following COVID infection, we did not include an induction interval and simply specified the 90-day window preceding a documented infection as the baseline interval and the 90-day window after infection as the subsequent interval. We note that we have previously applied a similar design in a pharmacovigilance context using the terminology of "sequence-symmetry analysis",[5] however, given that the current study is focused on effects of acute disease events as opposed to pharmacovigilance, we believe that the context makes the current terminology of "exposure-crossover design" more appropriate for this study. The 90-day duration was selected based on: (i) need for a time window that was sufficiently brief so that the exposure (i.e. COVID infection) and outcome (i.e. new medical diagnosis) could be temporally related, while also a sufficiently long so that an outpatient diagnosis could be identified; (ii) need for a time window that could minimize the possibility of confounding by one exposure type when considering effects of the other (i.e. given that vaccination administered after infection provides

© 2023 Kwan AC et al. *JAMA Network Open.*

an added immune-inflammatory challenge, and vice versa); and, (iii) the typical recommendation for most of the pandemic was to wait 90 days after infection to receive vaccination. The resultant self-controlled design effectively parallels the methodology commonly used in self-controlled acute exposure studies that have also leveraged medical care databases.[7] The design of the current study was specifically intended to control for multiple potential confounders related to healthcare engagement, given that differences in healthcare engagement in the baseline and subsequent intervals would affect accurate estimation of risk associated with infection. To further account for potential temporal confounders that may have been present during pandemic, though not directly related to individual-level COVID infection status, we identified new diagnoses of 'benchmark' medical conditions against which new diagnoses of cardiometabolic conditions could be compared (e.g. given the possibility of greater attention to health issues arising after compared to before having developed a COVID infection). Thus, the current study was designed to offer a relatively conservative approach to estimating the magnitude of risk for developing cardiometabolic disease after, compared to before, having sustained a SARS-CoV-2 infection.

Statistical Analyses

Using the exposure-crossover design,[3] we estimated crude odds of post- versus pre-infection diagnoses of diabetes, hypertension, hyperlipidemia, and benchmark diagnoses. We then constructed logistic regression models to estimate the odds ratio (OR) of new cardiometabolic diagnoses relative to benchmark diagnoses occurring during the post-COVID versus pre-COVID period while adjusting for age, sex, timing of index infection (pre-Omicron vs Omicron era), and pre-infection vaccination status. The multivariable logistic regression model was designed with the outcome being new diagnosis occurring within 90 days after infection versus before infection, with the key predictor being a new cardiometabolic disease diagnosis (diabetes, hypertension, hyperlipidemia) versus a new benchmark diagnosis. For a new cardiometabolic diagnosis occurring during the post-COVID versus pre-COVID time window, we also used multiplicative

© 2023 Kwan AC et al. *JAMA Network Open.*

terms to test for any possible interaction by age, sex, timing of infection being during pre-Omicron or Omicron era, or prior vaccination status. Statistical analysis was performed using R (v4.2.1, R Foundation for Statistical Computing), RStudio (v2022.07.1, RStudio PDC), with packages tidyverse (v1.3.1), janitor (v2.1.0), and lubridate (v1.8.0), with statistical significance defined as a two-tailed $p<0.05$.

© 2023 Kwan AC et al. *JAMA Network Open.*

eTable 1. Outpatient Diagnostic Codes Used for Analyses. The ICD codes, associated with outpatient visits, that were used to identify incident diagnoses of diabetes, hyperlipidemia, hypertension, and the benchmark conditions (urinary tract infection and gastroesophageal reflux disease) are shown below.

**Diagnosis**	**ICD-9**	**ICD-10**
Hyperlipidemia	272.2, 272.4,	E78.2, E78.4, E78.5
Hypertension	401.*, 402.*, 403.*, 404.*, 405.*	I10.*, I11.*, I12.*, I13.*, I15.*
Diabetes	250, 250.00, 250.0, 250.1, 250.2, 250.3, 250.4, 250.5, 250.6, 250.7, 250.8, 250.9,	E10.0, E10.1, E10.2, E10.3, E10.4, E10.5, E10.6, E10.7, E10.8, E10.9, E11.0, E11.1, E11.2, E11.3, E11.4, E11.5, E11.6, E11.7, E11.8, E11.9, E12.0, E12.1, E12.2, E12.3, E12.4, E12.5, E12.6, E12.7, E12.8, E12.9, E13.0, E13.1, E13.2, E13.3, E13.4, E13.5, E13.6, E13.7, E13.8, E13.9, E14.0, E14.1, E14.2, E14.3, E14.4, E14.5, E14.6, E14.7, E14.8, E14.9
Urinary Tract Infection	595.0, 599.0	N39.0
Gastroesophageal Reflux Disease	530.1, 530.10, 530.12, 530.19, 530.81	K21, K21.9

© 2023 Kwan AC et al. *JAMA Network Open.*

eReferences

1. Joung SY, Ebinger JE, Sun N, Liu Y, Wu M, Tang AB, Prostko JC, Frias EC, Stewart JL, Sobhani K, et al. Awareness of SARS-CoV-2 Omicron Variant Infection Among Adults With Recent COVID-19 Seropositivity. *JAMA Netw Open*. 2022;5:e2227241. doi: 10.1001/jamanetworkopen.2022.27241
2. Wei W-Q, Bastarache LA, Carroll RJ, Marlo JE, Osterman TJ, Gamazon ER, Cox NJ, Roden DM, Denny JC. Evaluating phecodes, clinical classification software, and ICD-9-CM codes for phenome-wide association studies in the electronic health record. *PloS one*. 2017;12:e0175508.
3. Redelmeier DA. The exposure-crossover design is a new method for studying sustained changes in recurrent events. *Journal of Clinical Epidemiology*. 2013;66:955-963. doi: https://doi.org/10.1016/j.jclinepi.2013.05.003
4. Braeye T, Hens N. Optimising the case-crossover design for use in shared exposure settings. *Epidemiol Infect*. 2020;148:e151. doi: 10.1017/s0950268820000916
5. Kwan AC, Ebinger JE, Wei J, Le CN, Oft JR, Zabner R, Teodorescu D, Botting PG, Navarrette J, Ouyang D, et al. Apparent risks of postural orthostatic tachycardia syndrome diagnoses after COVID-19 vaccination and SARS-Cov-2 Infection. *Nature Cardiovascular Research*. 2022. doi: 10.1038/s44161-022-00177-8
6. Maclure M. The case-crossover design: a method for studying transient effects on the risk of acute events. *Am J Epidemiol*. 1991;133:144-153. doi: 10.1093/oxfordjournals.aje.a115853
7. Takeuchi Y, Shinozaki T, Matsuyama Y. A comparison of estimators from self-controlled case series, case-crossover design, and sequence symmetry analysis for pharmacoepidemiological studies. *BMC medical research methodology*. 2018;18:1-15.

© 2023 Kwan AC et al. *JAMA Network Open.*
