# Supplementary material for: Association of COVID-19 Vaccination With Risk for Incident Diabetes After COVID-19 Infection
Source: JAMA Netw Open. 2023 Feb 14;6(2):e2255965. doi: 10.1001/jamanetworkopen.2022.55965 (PMC9929690; doi:10.1001/jamanetworkopen.2022.55965)
Supplement: Supplement 2. — Data Sharing Statement [file jamanetwopen-e2255965-s002.pdf]

Data Sharing Statement

Kwan. Association of COVID-19 Vaccination With Risk for Incident Diabetes After COVID-19 Infection. *JAMA Netw Open*. Published February 14, 2023.
doi:10.1001/jamanetworkopen.2022.55965

Data
Data available: No

Additional Information
Explanation for why data not available: Requests for de-identified data may be directed to the corresponding authors (AK and SC at biodatacore@cshs.org) and will be reviewed by the Office of Research Administration at Cedars-Sinai Medical Center prior to issuance of data sharing agreements, which are designed to ensure patient and participant confidentiality.
